# Dataset on fatal road traffic crash attributes extracted via natural language processing of online media articles in India

**DOI:** 10.1016/j.dib.2025.111578

**Published:** 2025-04-23

**Authors:** Ashutosh Ashutosh, Sai Chand

**Affiliations:** Transportation Research and Injury Prevention Centre (TRIP Centre), Indian Institute of Technology Delhi, Hauz Khas, New Delhi 110016, India

**Keywords:** Road safety, Traffic crash fatalities, News reporting, Crash data

## Abstract

Road traffic crashes are among the leading causes of death globally, resulting in substantial social and economic impacts. Online media is a key source of public information on road safety. Understanding how crashes are reported is crucial for detecting potential reporting biases and enhancing safety awareness. Hence, to address the issue of the lack of high-quality, media-reported fatal crash data, fatal crash reports were extracted for 2022–2023 from The Times of India, a prominent Indian news outlet. The resulting dataset comprised 2898 fatal crashes, 6584 fatalities and 7812 injuries, including 16 detailed crash attributes. This dataset was developed using web scraping and natural language processing (NLP) techniques. Automated tools such as Selenium and BeautifulSoup were employed to extract raw data from the news source. NLP algorithms were then applied to identify key crash attributes, including crash date, location, vehicles involved and number of fatalities. This study provides a replicable framework for constructing robust datasets from media sources, enabling multidisciplinary research on transportation safety, media reporting and public perception of crashes. The dataset is expected to serve as a valuable resource for analysing how the media shapes road safety narratives and for investigations on identifying high-fatality crash locations.

Specifications Table

**Table:** 

Subject	Safety Research
Specific subject area	Fatal Road Traffic Crashes, Automated Data Extraction, Natural Language Processing
Type of data	Processed Tabular Crash Attributes Data Unprocessed Raw Road Traffic Crash Articles Data Analysis Python Code Files
Data collection	Articles on fatal road traffic crashes (RTCs) published in The Times of India were collected using Python programming. Furthermore, the Selenium package within Python was used to automate webpage handling for identifying relevant fatal RTC articles. Frequently used keywords from RTCs were applied to identify 90 % of the fatal RTC articles published on the website. Subsequently, the BeautifulSoup Python package was used to determine the HTML tags that contained the article’s title, publication date and entire content, and these articles were extracted. The Natural Language Toolkit package was used to extract the relevant crash attributes from the raw article data. This process yielded the final dataset, which included comprehensive details of attributes for fatal RTCs.
Data source location	Country: India
Data accessibility	Repository name: Mendeley Data Data identification number: 10.17632/bc5sv6wnd9.7 Direct URL to data: https://data.mendeley.com/datasets/bc5sv6wnd9/7
Related research article	None

## 1 Value of the Data

• This dataset provides unique insights into fatal road traffic crashes (RTCs) that have garnered considerable media attention in India, highlighting potential biases in media coverage. Such insights are not obtained from conventional crash datasets. Researchers can use this dataset to explore the specific narratives employed by The Times of India, a prominent Indian news source, when reporting crashes deemed significant in the national context.
• This dataset includes both qualitative and quantitative attributes of RTCs, specifically from media-reported sources within the country, permitting the identification of factors that influence the likelihood of a fatal crash being reported by this specific media source. However, generalisations beyond the studied country and the specific media outlet require additional research.
• This dataset is valuable for determining crash locations in India associated with high fatalities, as reported by the media. Spatial and temporal analyses of these locations could help authorities pinpoint and address specific infrastructure safety issues. Additionally, qualitative data can aid in studying reporting patterns, biases and the sentiments expressed by editors and writers in their coverage of fatal crashes. These data can support the development of Large Language Models to analyse media communication patterns and suggest improvements in road safety reporting.

## 2 Background

According to the World Health Organisation (WHO), millions of people die annually owing to road traffic crashes (RTCs), which are the 12^th^ leading cause of death among adults and the leading cause among children and young adults aged 5–29 years. Approximately two-thirds of these fatalities involve the working-age population (18–59 years), resulting in considerable social and economic impacts [1]. Low- and middle-income countries experience disproportionately high outcomes from RTCs, with fatality rates nearly three times higher than those in high-income countries. India has implemented several road safety initiatives, including amendments to the Motor Vehicles Act and increased funding for state-level safety programmes [2]. Nonetheless, contrary to the WHO’s Sustainable Development Goal of reducing road traffic fatalities by 50 % by 2030 [1], India has witnessed a continued escalation in fatalities, with a compound annual growth rate of 1.7 % between 2018 and 2022 [3]. These statistics highlight the deeply ingrained nature of the road safety challenge in India and underscore the need for a comprehensive strategy to enhance public awareness. Effective public communication could augment support for more decisive policy interventions and safer behavioural norms, aligning the country with its sustainable development goals.

Media serves as a primary source of public information and communication; therefore, understanding the link between media coverage and traffic safety awareness is critical. Research has shown that limited reporting of traffic crashes is associated with fewer policy reforms and that adequate media attention can enhance public support for road safety initiatives [4,5]. Although the media plays an essential role in promoting road safety [6], editorial decisions on which incidents to report often reflect organisational interests rather than public education goals [7]. Disproportionate media coverage of crashes involving youngsters may lead to overestimations of youth fatalities, distorting public perception and behavioural responses towards actual crash trends and vulnerable populations [8].

Media reports inherently provide selective coverage of crashes, prioritising incidents perceived as newsworthy. Hence, systematic underreporting occurs, and the resulting datasets represent only a fraction of the actual fatal RTCs. This fundamental issue introduces biases and must be considered when interpreting the findings. Investigating the biases introduced by media reporting can, therefore, reveal key factors that influence public understanding of road safety. Nevertheless, the lack of comprehensive datasets on media-reported fatal RTCs limits researchers’ ability to explore such biases. This data article aims to bridge this gap and draw attention to an under-investigated yet impactful area of research—the role of media in transportation system safety*.*

## 3 Data Description

The data are present in the Mendeley Data Repository entitled ‘Media-Reported Road Traffic Crash Data’ [9]. This repository includes three folders. The ‘Data Files’ folder contains the actual dataset and the data dictionary explaining the dataset; the ‘Data Extraction and Analysis Codes’ folder contains the codes for data extraction, data fusion and extraction of crash variables from raw data; and the ‘Raw Data’ folder contains raw CSV files possessing raw data in the form of news articles.

### 3.1 The ‘Data Files’

The ‘Data Files’ folder contains the dataset and the data dictionary. This dataset includes crash data reported by The Times of India (TOI) for 2022 and 2023, comprising 16 variables per crash. The data dictionary file provides the names, data types and descriptions of these variables. According to the guidelines of the Permanent International Association of Road Congress, a non-profit organisation striving to enhance road and transport infrastructure globally [10], the following attributes were identified to be most significant:

1. Crash Identification Number
2. Accurate Crash Location Name
3. Type of the Crash
4. Information on Victims
5. Information on Vehicles
6. Information on Crash time
7. Information on Weather

Based on these inputs, the following crash attributes were selected: Crash Number, Month, Crash Date, Crash Day, Location, Million-Plus City, State, LatLong, Vehicle 1, Vehicle/Object 2, Killed, Injured, Age, Gender, Road Type and Crash Type, i.e. 15 attributes. Furthermore, complete weather information was missing from the articles; therefore, this attribute was excluded. The Article Date attribute was added as news articles served as crash data sources. Table 1 provides a brief description of each variable. The dataset contains 2898 fatal RTCs. These crashes resulted in 6584 fatalities and 7812 injuries. The following sections discuss the attributes included in the data, illustrated via figures and deductions.

**Table 1:** Crash attributes description.

S. No.	Attribute name	Description	Data type
1	Crash Number	A unique ID, given to each crash.	Integer
2	Month	This attribute contains the month in which the crash occurred.	Character
3	Crash Date	This attribute contains the date on which the crash occurred.	Date
4	Crash Day	This attribute contains the day of the week on which the crash occurred.	Character
5	Article Date	This attribute contains the date the article was published for the crash.	Date
6	Location	This attribute contains the crash location name.	Character
7	Million Plus City	This attribute contains the million-plus population city name if the crash happened within its administrative boundary.	Character
8	State	This attribute contains the name of the state in which the crash occurred.	Character
9	LatLong	This attribute contains the coordinates of the location where the crash happened.	Tuple
10	Vehicle 1	This attribute contains the vehicle type of crash victims.	Character
11	Vehicle/Object 2	This attribute contains the other vehicle/object involved in the crash.	Character
12	Killed	This attribute contains the total number of individuals who died in that crash.	Integer
13	Injured	This attribute contains the total number of injured individuals in the crash.	Integer
14	Age	This attribute contains the age of the victims who died in the crash.	String
15	Gender	This attribute contains the gender of individuals who died in the crash.	Character
16	Road Type	This attribute contains the type of road on which the crash occurred.	Character
17	Crash Type	This attribute contains the collision type.	Character

**Fig. 1:**
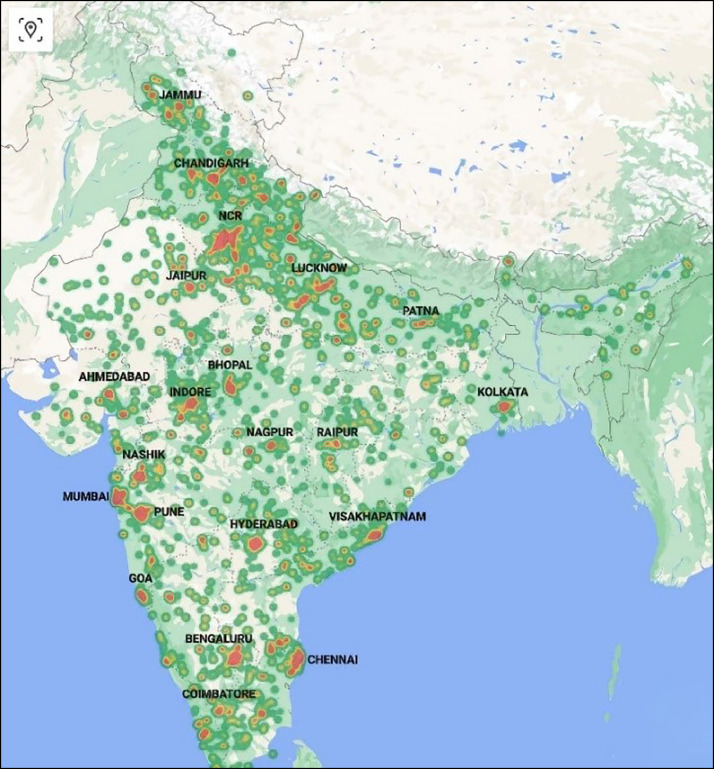
Heatmap of high fatality crash locations.

Fig. 1 presents a heatmap of fatal crash locations, with red clusters indicating cities with the highest number of media-reported fatalities. The largest ones correspond to major metropolitan areas with populations exceeding one million, as labelled on the map. The most prominent cluster is the National Capital Region.

**Fig. 2:**
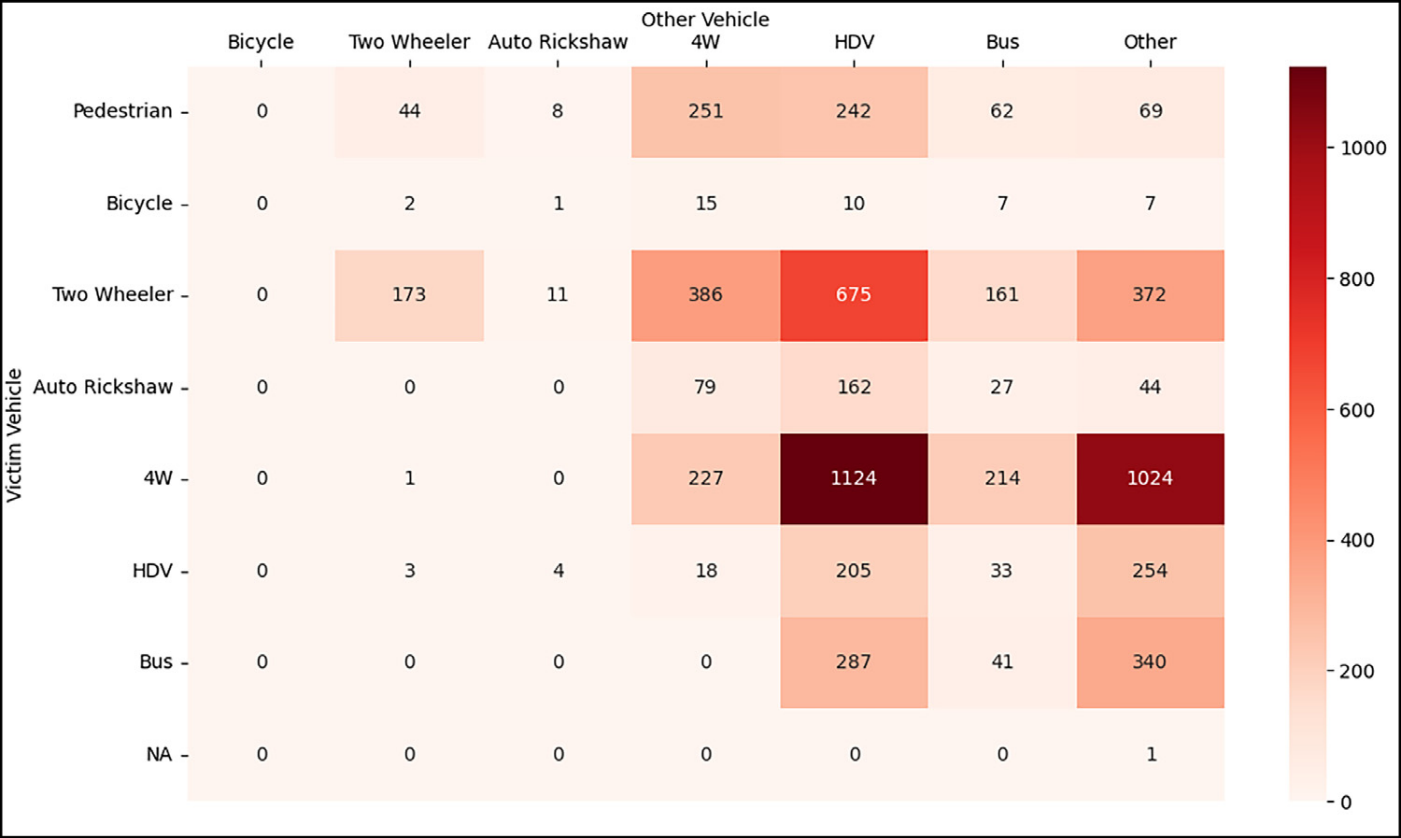
Who-hit-whom matrix between victim and striking vehicle type.

Fig. 2 illustrates the ‘who-hit-whom’ matrix derived from the dataset, displaying the distribution of fatalities by victim and striking vehicle types. Four-wheelers (4Ws), including SUVs, vans, cars and ambulances, are grouped under a single category. Heavy-duty vehicles (HDVs) include trucks, tractors, dump trucks, earthmovers and excavators. The ‘others‘ category primarily comprises stationary and non-vehicular objects such as road dividers, poles, trees, gorges, canals, unidentified vehicles, walls and culverts. The matrix reveals that the highest fatalities occurred in crashes where HDVs struck 4Ws.

**Fig. 3:**
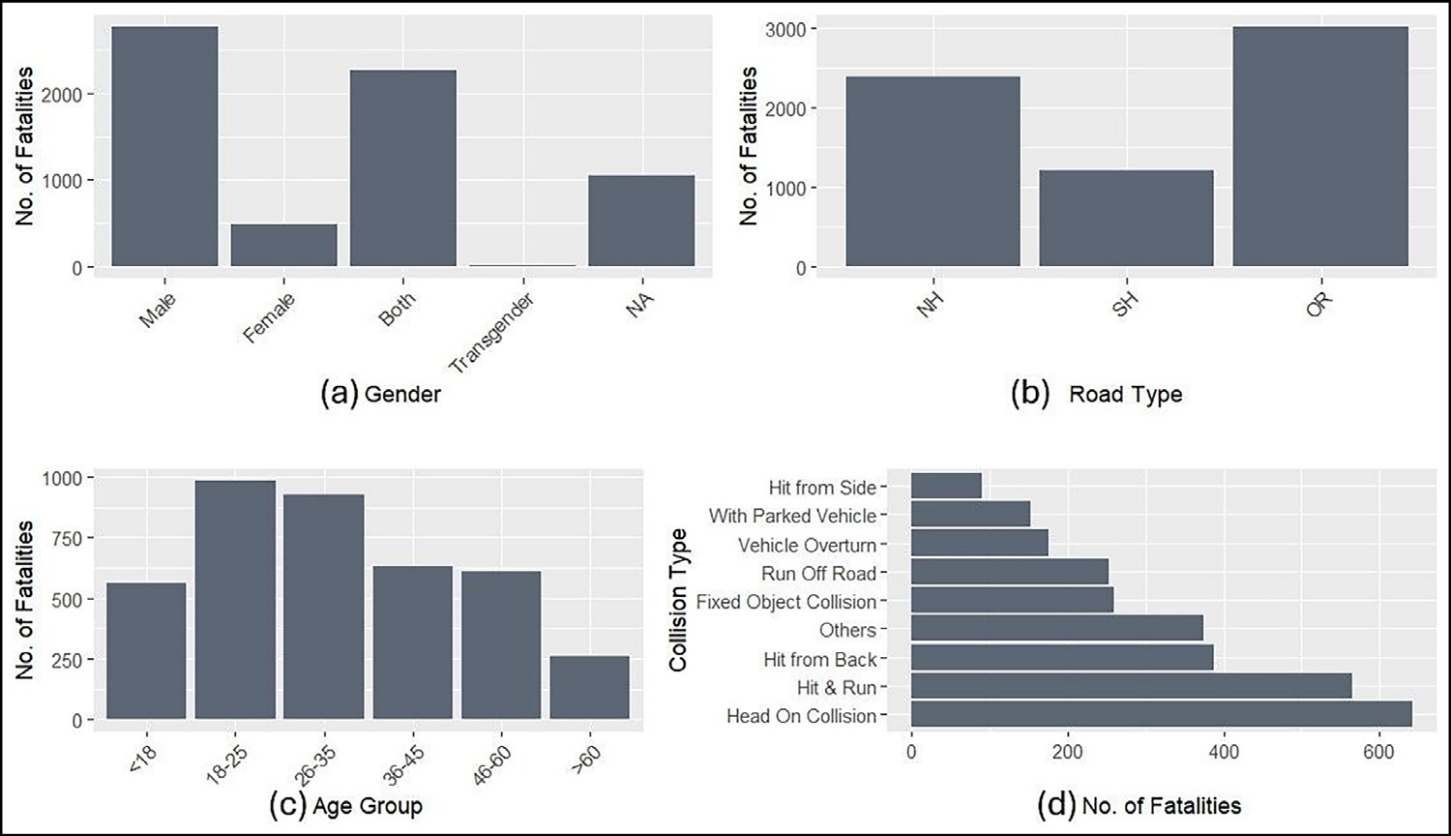
Fatalities distribution among different attributes.

Fig. 3 presents the frequency distribution of fatalities across gender, road type, age group and collision type. The gender-wise distribution in Fig. 3(a) indicates that most fatalities involved male victims. Fig. 3(b) shows that the road type also played a key role, with 2379 fatalities occurring on national highways, 1199 on state highways and 3006 on other roads, including residential streets, city roads, village roads and other minor classifications. The age-wise distribution in Fig. 3(c) signifies that the highest number of fatalities involved individuals aged 18–25 years, whereas the lowest involved those aged over 60 years. Finally, Fig. 3(d) illustrates the distribution of fatalities by collision type, as reported in the news, highlighting that head-on collisions, hit-and-run incidents and rear-end collisions are among the most commonly reported ones and are associated with high fatalities.

### 3.2 The ‘Data Extraction and Analysis Codes‘

The ‘Data Extraction and Analysis Codes‘ folder contains three Python files. The ‘Data Extraction.py‘ file extracts raw data from the webpage. The ‘Combined Month Data.py‘ file combines the raw data files extracted over a month using ‘Data Extraction.py‘. Ultimately, the ‘Crash Variables Extraction.py‘ file extracts the crash attributes from the raw file containing a month’s data. The ‘Experimental Design, Materials and Methods' section provides a detailed explanation of each step in the data collection process.

### 3.3 The ‘Raw Data‘

The raw data folder contains two sub-folders, one for 2022 and the other for 2023. Each folder contains 12 CSV files, one for each month of the year, in the specified format. In addition, two CSV files, one for each year, are named ‘2022_raw_data‘ and ‘2023_raw_data’. These contain the complete raw data for each year, obtained by merging the individual monthly files. Table 2 presents a sample structure of the raw data file, including Article Title, Article Date and Article Content.

**Table 2:** Sample raw data entry.

Title	Date	Content
63-yr-old killed in road accident	Aug 3, 2023, 08:28 IST	Nashik: A motorist died in a road accident when his vehicle was knocked down by a tempo at Nashik Road on Tuesday. The victim, Dinkar Tajanpure (63), was riding his motorcycle on Nashik Road on Monday evening when a tempo coming from behind allegedly hit him, causing him to fall. The driver fled the scene. A complaint over causing death due to negligent driving was registered with Nashik Road police station“Tajanpure’s condition was critical. He received grievous injuries in his head and chest. He succumbed to treatment while being in the hospital,” said an officer. The police said that though the motorist had fled the scene along with the vehicle, CCTV footage would help track him. “We have various organisations on the road whose CCTV footage is being used to track the vehicle's details and reach the motorist,” the officer said.

This raw dataset holds immense value for academic researchers, particularly those involved in media analysis, transportation safety and public perception. This dataset can be leveraged for sentiment analysis for examining community reactions to fatal crashes, as reflected in news articles. Furthermore, the tone of reporting can be assessed to determine whether the language used by editors tends to be sensationalised—emphasising drama and emotion—or focused on safety and accountability issues. Moreover, researchers can compare these media reports with First Information Reports filed by the police to assess the extent to which sensationalism versus fact-based reporting is employed in covering fatal crashes.

## 4 Experimental Design, Materials and Methods

This section outlines the methodologies used to gather raw data and subsequently filter them to obtain complete crash attributes from news-reported fatal RTCs.

### 4.1 Data source identification

The data for this research were collected from the online news archives of TOI. According to the figures compiled by the Media Research Users Council in the Indian Readership Survey Q4 2019, TOI was the most-read English-language newspaper in India and the ninth most-read newspaper among all language newspapers [11]. A rigorous analysis was conducted to determine whether the fatal RTC data collected from TOI could effectively represent the crash information conveyed to the public by the media. For reliable national estimates, our chosen news source must meet the following criteria, and TOI performed well in all these criteria:

1. Broad National Coverage and High Readership
2. Well-Maintained Archive of Past Articles

### 4.2 Broad national coverage and high readership

Based on a 2024 Statista report, 77 % of the global population accesses news via smartphones, making online media the most widely consumed news source globally [12]. In India, nearly 70 % of the population consumes news via digital platforms [13]. According to the World Association of Newspapers and News Publishers, TOI is the world’s largest circulated English-language daily across all formats, including broadsheet, compact, Berliner and online editions [14]. Verified by the Audit Bureau of Circulations, the readership of TOI exceeds 5.5 million, making it the most-read English-language newspaper in India and one of the top three online news sources for Indian audiences [15]. Furthermore, the India Digital News Report by the Reuters Institute ranks TOI as the most trusted news brand among Indian media outlets [16]. These figures highlight TOI’s extensive reach, high credibility and relevance as a source for analysing media-reported RTCs.

### 4.3 Well-maintained archive of past articles

TOI maintains a well-structured digital archive of its published articles, organised by publication date, making it more accessible for research than other online news platforms. This archive dates back to January 2001, providing a comprehensive historical source of media-reported events [17].

While some may question whether TOI is more representative than regional news sources, available evidence suggests it is. TOI reaches approximately 70–75 % of individuals who consume news online on a daily basis. Although regional newspapers may enjoy higher physical circulation in specific linguistic regions, they often lack the digital presence and nationwide reach required for constructing a comprehensive dataset. Moreover, the digital archiving of Indian regional-language newspapers is often limited owing to lower demand and funding constraints compared with national-level sources such as TOI [18,19].

In India, 22 languages are officially recognised, and each one is primarily spoken in a specific geographic region. However, English, widely adopted as a second language, is increasingly prevalent across all states. A study by David Graddol, as outlined in English Next India [20], highlights the growing role of English in academia, the workplace and public life. Public signages across Indian states commonly include English translations, reinforcing its accessibility. English-language news articles offer broad comprehensibility for readers nationwide. In contrast, local-language news outlets often lack the digital infrastructure and cross-regional accessibility needed for large-scale media-based research. Therefore, TOI was selected as the primary data source owing to its extensive national reach, substantial online readership, consistent digital archiving and the fact that it is published in a language widely understood by the population.

### 4.4 Identification of keywords

After identifying the data source, appropriate keywords for article retrieval were determined. This process began by estimating the total number of articles typically published on the TOI website in a month. The content was manually analysed for a randomly selected month—June 2023—to evaluate the proportion of articles related to RTCs and road safety. A total of 14,715 articles were published in this month, of which 423 (3 %) focused on RTCs or road safety.

Of these 423 articles, 247 (58 %) specifically addressed fatal RTCs, 54 (12 %) concentrated on non-fatal RTCs, and the remaining 122 (29 %) covered general road safety topics. A term frequency analysis was subsequently performed on the titles of the 247 fatal RTC articles to identify commonly used keywords. The most frequently encountered terms were ‘Road‘, ‘Accident’, ‘Killed’, ‘Dead‘, ‘Dies‘ and ‘Death‘. These keywords were then tested in various combinations to assess their retrieval effectiveness.

This keyword set successfully retrieved 220 of the 247 fatal RTC articles, representing a retrieval accuracy of approximately 90 %. Given their high recall rate, these keywords were selected for automated data collection from the TOI website.

### 4.5 Raw data extraction

Python programming was used to automate data collection and filter out relevant crash attributes from the raw data, including the number of fatalities, injuries and crash location. To achieve this purpose, multiple Python packages were utilised to automate data collection. The following are some of the packages utilised:

• **Selenium:** The Selenium Package was applied to automate data collection from web browsers. This package is a powerful tool for automating web browsers, permitting users to programmatically simulate interactions with webpages. Selenium allows users to automate tasks such as form submission, button clicks, navigation and more complex tasks, such as testing and web scraping, with browsers such as Google Chrome, Firefox and Safari [21]. In our context, Selenium was used to open a web browser and access TOI webpages containing crash articles for a single day, collect all article-related data and then terminate the session to open a new one with the next day’s articles.
• **BeautifulSoup:** The BeautifulSoup Package is a tool for web scraping and parsing HTML and XML documents and webpages. This package efficiently navigates and modifies the content in webpages [22]. In our context, BeautifulSoup was used to identify HTML tags of webpage containers that included article headings, publication dates and article content and subsequently transmit the information to a data frame storing raw data.
• **NLTK:** The Natural Language Toolkit (NLTK) is an open-source Python Package that performs natural language processing (NLP) tasks. This package provides a wide range of functions to work with English-language tasks, such as tokenisation, lemmatisation, part-of-speech tagging and named entity recognition [23]. In our context, NLTK part-of-speech tagging and keyword recognition were used to identify crash attributes from the article content.
• **Pandas:** The Pandas Package is a data analysis tool that can efficiently transform, clean and analyse numerical and textual data. This package is designed to manage missing data, edit and reorganise datasets and simplify data alignment and merging tasks. In our context, its data frame functionality was utilised to store the article title, date and content in a data frame when multiple days’ data were collected. It combined these into one-month data [24].

**Fig. 4:**
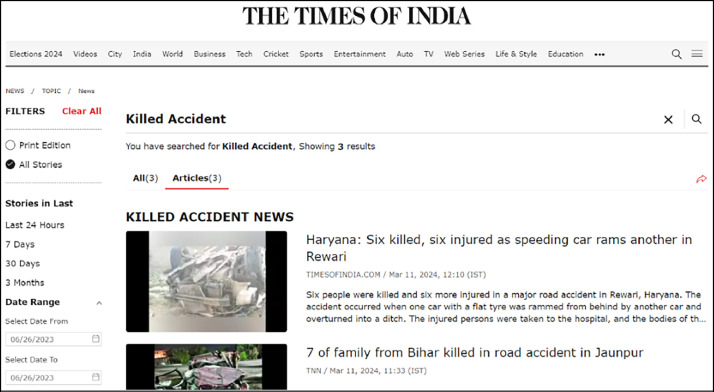
Raw data extraction interface of single-day fatal crash articles.

Using the identified keywords, relevant articles were filtered out from all articles published each day. Fig. 4 illustrates a sample interface that filters articles based on keywords for a single day.

Subsequently, the process of raw data collection began, with Selenium automatically opening the URL of the first article, which was identified based on its title using BeautifulSoup’s HTML container tags. The container tag of the article title was determined to be ‘#storyBody > div > div.crmK8 > div > div > div > div > div > div:nth-child({i}) > a > div > div.fHv_i.o58kM > span‘.

**Fig. 5:**
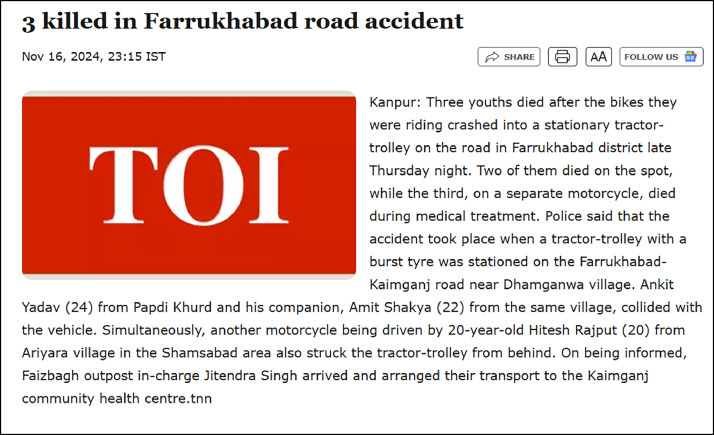
Sample article content interface containing crash attribute details.

Here, ‘i‘ represents the serial number of the article title out of all the articles on the webpage. For instance, if there were six articles on a given day, each article title had the same container tag, differentiated by the ‘i‘, whose value ranged from 1 to 6. Then, Selenium clicked on the hyperlink of the first news article on the webpage to land on the page containing its entire content, a sample of which is illustrated in Fig. 5.

On this page, BeautifulSoup again extracted the article title using the HTML container tag ‘div.NvaTO > div.cCU6C.innerbody > div.okf2Z > div.pZFl7 > h1 > span‘ and the date using the tag ‘div.NvaTO > div.cCU6C.innerbody > div.okf2Z > div.pZFl7 > div > div.xf8Pm.byline > span‘. The same container tags were used for all article titles and publication dates. However, different tags were used for extracting the content. A comprehensive list of 11 container tags was prepared, and BeautifulSoup was used to loop through it, matching the tags with the article content container and extracting the relevant information. One such sample article content container tag is ‘/ html / body / div [2] / div / div [3] / div [3] / div [2] / div [1] / div [1] / div [5] / div [2] / div / div [2] / div [2]‘. Other container tags also exhibited the same structure; only the integer values in div[i] were in an unorganised sequence, containing any digit from 0 to 9. The code opened an article and then used BeautifulSoup to compare the listed container tags with those on the webpage. When a successful match was obtained, it retrieved and saved the textual content within these tags into a data frame. Selenium continued this process until each article’s information was successfully extracted.

Following the keyword identification process, data were curated daily for one month. Raw RTC articles were collected for each day and stored in separate CSV files, which were then aggregated to create a comprehensive monthly dataset. Nonetheless, some non-relevant articles were also captured during this process. These articles did not report on fatal RTCs, although they contained the selected keywords in their titles. Instead, many featured political statements or commentary on road safety, lacking substantive information about specific crash events.

Such irrelevant articles were manually reviewed and removed from the dataset to ensure the quality of the data. This cleaning step was necessary to retain only those articles that reported actual fatal RTCs. The same procedure was followed for each month’s data, resulting in the complete raw dataset, which is provided in the ‘Raw Data‘ folder.

### 4.6 Crash attributes extraction

This stage involved extracting detailed crash attributes from the monthly raw dataset. Of all the attributes, only the ‘Article Date‘ field was in a directly usable format. A unique identifier labelled ‘Crash Number‘ was assigned to each crash instance during the extraction process and did not require parsing from the article content. The ‘Month‘ attribute was derived from the ‘Article Date‘, as the date string included the month name. The remaining attributes were extracted using rule-based NLP methods, guided by recurring patterns in article formatting. The content shown in Fig. 5 is used as a representative example to illustrate the process, as its structure is typical of most fatal RTC articles published by TOI.

To extract the ‘Crash Day‘ attribute, a keyword search method was applied to detect the presence of weekday terms (e.g. Monday to Sunday) in the article text. For instance, in the sample article, the identified value was ‘Thursday‘. This basic rule-based approach was effective owing to the frequent inclusion of the crash day in reporting. The HeidelTime Python package [25] was employed for ‘Crash Date‘ extraction using the article’s publication date and the crash day as input parameters. HeidelTime interprets temporal expressions in context, allowing it to estimate the precise date of the crash. In the example, with an article date of 16 November 2024 and a reported crash day of Thursday, HeidelTime correctly inferred the crash date.

The ‘Location‘ attribute was extracted via pattern recognition using keyword-based rules. Terms such as ‘near,‘ ‘at‘ and ‘in‘ were used to identify phrases likely to contain location names. Furthermore, a consistent pattern was observed, with the city name appearing at the beginning of TOI articles. These initial city mentions were also extracted to enhance location coverage. The algorithm searched for ‘Road‘ to capture road names and extracted the two preceding words as these typically denoted the road’s name. Potential location candidates identified in the example article were ‘Farrukhabad‘, ‘Dhamganwa‘, ‘Farrukhabad–Kaimganj road‘ and ‘Kanpur‘. Of these, ‘Dhamganwa‘, the one most relevant at the local crash level, was selected as the final ‘Location‘ attribute.

Crash location names extracted from the article content were input into the Google Maps Geocoding API [26] to obtain the corresponding geographic coordinates (latitude and longitude). Although this automated method was generally effective, it did not recognise certain location names mentioned in the articles. In such cases, a manual search was conducted using Google Maps [27], which enabled the successful retrieval of coordinates. For instance, in the example case, this process yielded the coordinates (27.515, 79.405).

To assign the ‘Million-Plus City‘ attribute, each crash location’s coordinates were manually checked against the administrative boundaries of Indian cities with populations exceeding one million [28]. The attribute was assigned as ‘Nil’ if the coordinates fell outside any such boundary. In the example case, the location did not correspond to a million-plus city, so ‘Nil‘ was recorded. Similarly, the crash coordinates were used to identify the state in which the crash occurred. A manual verification process ensured that the ‘State‘ attribute was accurately assigned. In the given example, the crash occurred in the state of Uttar Pradesh.

The ‘Victim’s Vehicle‘ (Vehicle 1) and ‘Striking Vehicle/Object‘ (Vehicle 2) attributes were extracted using a rule-based keyword-matching algorithm tailored to the narrative structure of fatal RTC articles. This algorithm initially scanned the article content for the earliest mention of a vehicle from a predefined list and assigned it as Vehicle 1. This name was deleted from the text, and the search was repeated to identify a second vehicle, designated as Vehicle 2. In cases where both vehicles were of the same type, the second occurrence was not found, and Vehicle 2 was recorded as blank. This blank value implicitly indicated that both vehicles were of the same type. For example, in the sample case, Vehicle 1 would be recorded as ‘Bike‘ and Vehicle/Object 2 as ‘Tractor–Trolley‘.

To extract the ‘Killed‘ and ‘Injured‘ attributes, a keyword search algorithm was applied to the article titles. Terms such as killed, died and dead were used to identify fatalities, whereas keywords such as injured and injury were used to detect non-fatal injuries. For each case, the algorithm extracted the numeral appearing up to two words before the keyword in the title. If no mention of injuries was found, the injured field was left blank. In the example case, the title contained ‘3 killed‘, leading to a value of 3 for ‘Killed‘, and as there was no reference to injuries, a value of 0 was recorded for ‘Injured‘.

The ‘Age‘ attribute of fatal crash victims was extracted using a pattern-based approach, following a standard formatting convention used in the articles. Usually, the victims’ ages are mentioned in parentheses immediately after their names. Hence, the algorithm scanned the article content for numeric values enclosed in parentheses and aggregated these values into the ‘Age‘ field. This attribute specifically captured the ages of individuals who died in the crash, as information regarding the ages of injured victims was typically absent. If, for example, four fatalities were reported in an article, the corresponding entry in the age field would ideally contain four distinct values separated by commas (e.g. ‘24, 22, 20, 19‘). Nevertheless, owing to incomplete reporting, fewer age values may be recorded. In the example case, the extracted ages would be ‘24, 22, 20‘.

The ‘Gender‘ attribute was determined manually by analysing how victims were referenced in the article. Gender was inferred based on victim names as well as gendered pronouns and descriptors such as ‘he‘, ‘she‘, ‘his‘ and ‘her’. If all identified victims were male, the value ‘Male‘ was assigned, and if all were female, ‘Female‘ was recorded. If victims included individuals of both genders, the attribute was labelled ‘Both‘. In rare cases where the article explicitly stated that the victim was transgender, the attribute was marked as ‘Trans‘. In the example case, the victims were identified as male; therefore, ‘Male‘ would be assigned for the ‘Gender‘ attribute.

**Fig. 6:**
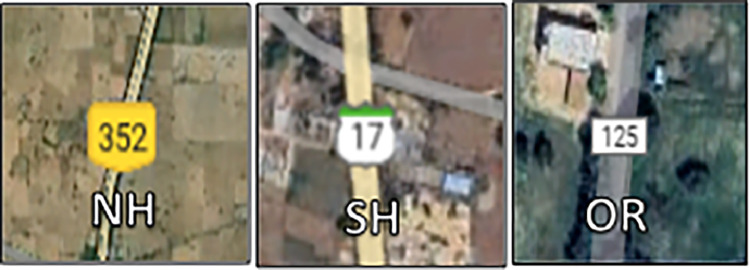
Representation of different road types on google maps.

The ‘Road Type‘ attribute was manually identified using Google Maps. Road classifications, such as national highways, state highways or other local roads, were determined by cross-referencing the crash location with the road labels on Google Maps. Fig. 6 illustrates the various road types designated on the platform. The identified road type was classified as ‘OR‘ (Other Roads) in the example case.

The ‘Crash Type‘ attribute was also manually extracted and supplemented with keyword-matching techniques. Specific terms within the article content, such as stationary, hit from behind, mowed down and similar expressions, were used to infer the nature of the collision. For example, the presence of the word stationary was interpreted as indicative of a ‘Fixed Object Collision‘. However, each identified crash type was verified by manually reviewing the article context to ensure accuracy.

### 4.7 Sanity checks and data validation

Experts in the field scrutinised and verified the dataset to ensure its accuracy. Multiple checks were performed on crash attributes to determine their quality.

Duplicate entries for a single crash were identified and eliminated to ensure that only unique entries were present in the crash attributes data. This process identified entries involving fatal crashes with identical victim vehicles, impacting vehicles, crash day and number of fatalities and subsequently highlighted such entries. A manual check was then performed to eliminate the duplicates. Duplicate entries could have been generated owing to multiple articles about the same crash being published on different days. Hence, the crash attribute data included only unique crashes.

Moreover, the coordinates of the crash location, which could not be extracted using the API, were obtained using a zeroing-in process. This process initially identified the state name, then the district name, and finally, the exact location or road name to pinpoint the crash site. Similar to the data validation exercise by Sinha et al [29], both authors performed this task independently and separately. The coordinates were subsequently matched to ensure consistency. This process ensured that the location coordinates were precise and reliable. Furthermore, the geocodes generated by the API were verified to confirm that their locations fell within the Indian boundary and the road section specified in the article. Hence, the dataset was checked for its quality and unbiasedness.

Ultimately, to ensure data quality, the results of previous research studies were reviewed to determine whether they matched our dataset. Our crash dataset has 2898 fatal crashes with 6584 fatalities; hence, the fatal crash severity, i.e. the number of people killed per crash, is 2.27; however, as per government-released data, the fatal crash severity is only 1.08 [3]. This difference emphasises that fatal crashes with higher fatalities are prioritised in reporting. For instance, De Ceunynck et al. observed that mean fatalities in media-reported fatal crashes were 1.2, as opposed to 1 in the official database for such crashes [30]. In addition, crashes occurring on a weekend receive higher media coverage than those on a weekday, which is also reflected in our data, with ‘Sunday‘ and ‘Saturday’ being the most common entries for the ‘Crash Day‘ variable. Ultimately, in terms of vehicle type involved in the crash, our data showed that ‘Cars‘, ‘Buses‘ and ‘Trucks‘ had a disproportionately higher representation in media-reported fatal crashes compared with 'Pedestrians‘, ‘Bicycles‘ and ‘Two-Wheelers‘. De Ceunynck et al. also observed that crashes involving buses and heavy goods vehicles have a five-fold and two-fold higher chance of being reported, respectively. In comparison, those involving powered two-wheelers (i.e. mopeds and motorcycles) have a significantly lower chance (i.e. one-fourth and half, respectively) of being reported. Crashes involving cyclists also have a lower probability of being reported. These checks and statistics highlight the rigorous processes adopted to verify data quality and the similarity of crash attributes in this dataset to those in others.

### 4.8 The stepwise sequence to analyse the dataset

To interact meaningfully with the dataset and fully leverage its potential, we recommend that readers follow the step-by-step procedure outlined below.

Step 1: Using the ‘Raw Data‘ folder in the repository, read and understand how the media writes an article on RTCs, an example of which is provided in Table 2. Here, you can notice which crash details are mentioned by the media.

Step 2: Use the ‘Data Extraction.py‘ code in the repository folder ‘Data Extraction and Analysis Codes‘ to understand the methods adopted by the authors to extract raw data. This step will help readers comprehend data collection and the details that can be used to replicate the process.

Step 3: Use the ‘Combined Month Data.py‘ code in the ‘Data Extraction and Analysis Codes’ repository folder to understand the methods used by the authors to combine daily files into monthly files. Raw data in the form of monthly records will facilitate further analysis.

Step 4: Use the ‘Crash Variables Extraction.py‘ code in the repository folder ‘Data Extraction and Analysis Codes‘ to understand the methods followed by the authors to extract relevant crash attributes from the raw data. All crash attributes can be extracted from the raw data using the methods of the article editors.

Step 5: This is the most important step. Read and analyse the ‘News Crashes.xlsx‘ file in the ‘Data Files‘ folder. These data represent the true value of our research; investigators can use them to enhance road safety research initiatives. The following are some of the specific analyses that researchers could conduct:

• Identifying blackspots: Researchers can perform spatial analyses to detect crash hotspots, especially in urban regions, and correlate these locations with regional demographics and road infrastructure characteristics. This process can help develop location-specific speed reduction measures to alleviate fatalities.
• Identifying Peak Crash Periods: Temporal analysis can identify specific months or holiday periods, such as the Christmas and New Year week, associated with elevated fatal crash rates. Such insights can guide lawmakers in augmenting the surveillance for traffic violations during these periods.
• Identifying Vulnerable Road Users: By exploring the patterns in vehicle types involved in fatal crashes, researchers can identify high-risk user groups and vehicle categories. These findings can inform the design of vehicle-specific safety measures or the implementation of targeted driver education and enforcement programmes.

### 4.9 Limitations and scope for future work

Although this dataset is of considerable value to the research community, certain limitations associated with its use should be acknowledged. The first limitation concerns potential underreporting, a common issue in several RTC datasets [31]. Media coverage inherently captures only a selective subset of road crashes, usually those with higher fatalities involving certain types of vehicles or those occurring under particular circumstances deemed newsworthy. Thus, this dataset does not reflect the complete spectrum of road traffic fatalities but highlights those selectively reported by the media.

The second limitation stems from the dataset’s reliance on a single media source (TOI). Although TOI enjoys broad national coverage and is highly representative at the macro level, it may not uniformly capture crash incidents across all regions of the country. Regional readership density, the availability of local reporters and the differences in urban vs rural coverage may affect the geographical distribution of reported crashes. Hence, the dataset may have limited representativeness for micro-level or region-specific analyses.

Additional data sources, such as regional or vernacular-language news outlets, could be integrated to enhance the comprehensiveness and regional representation of future versions of this dataset. Thereby, its applicability for both localised and comparative crash research across different parts of the nation could be strengthened.

## Ethics Statement

This study utilised crash data acquired from publicly accessible online sources. No human participants, animal subjects or information from social media platforms were involved in the data collection process. The dataset excludes personally identifiable or sensitive information while retaining victim attributes (e.g. name, age and gender) only when deemed relevant and permissible under ethical research guidelines. The study adhered to ethical research practices, emphasising transparency, reproducibility and integrity in data handling.

The authors also acknowledge using Grammarly for language editing, mainly to ensure the scientific clarity of written content. This data article documents a novel method for extracting crash attributes from online sources. While its statistical patterns are aligned with those of existing studies, the dataset has not yet been utilised in peer-reviewed research.

## CRediT Author Statement

**Ashutosh Ashutosh:** Methodology, Software, Validation, Formal analysis, Investigation, Resources, Data Curation, Writing - Original Draft and Visualisation. **Sai Chand:** Conceptualization, Methodology, Investigation, Resources, Writing - Review & Editing, Supervision and Project administration.

## Data Availability

Mendeley DataMedia Reported Road Traffic Crash Data (Original data).
